# Homemade magnet-assisted endoscopic removal of blades from a complex esophago-mediastinal fistula in a pediatric patient

**DOI:** 10.1055/a-2877-1913

**Published:** 2026-06-11

**Authors:** Xueqiang He, Yang Liu, Xianbao Zhou, Lin Wang, Zhongquan Zhu, Shengai Zhong, Dandan Guo

**Affiliations:** 1 Department of Gastroenterology 610828 The 924th Hospital of the People's Liberation Army Joint Logistics Support ForceGuilinChina; 2 Department of Thoracic Surgery 610828 The 924th Hospital of the People's Liberation Army Joint Logistics Support ForceGuilinGuangxiChina

A 13-year-old girl with childhood-onset emotional disorder presented with 7-hour chest pain after blade ingestion.

**Fig. 1:**
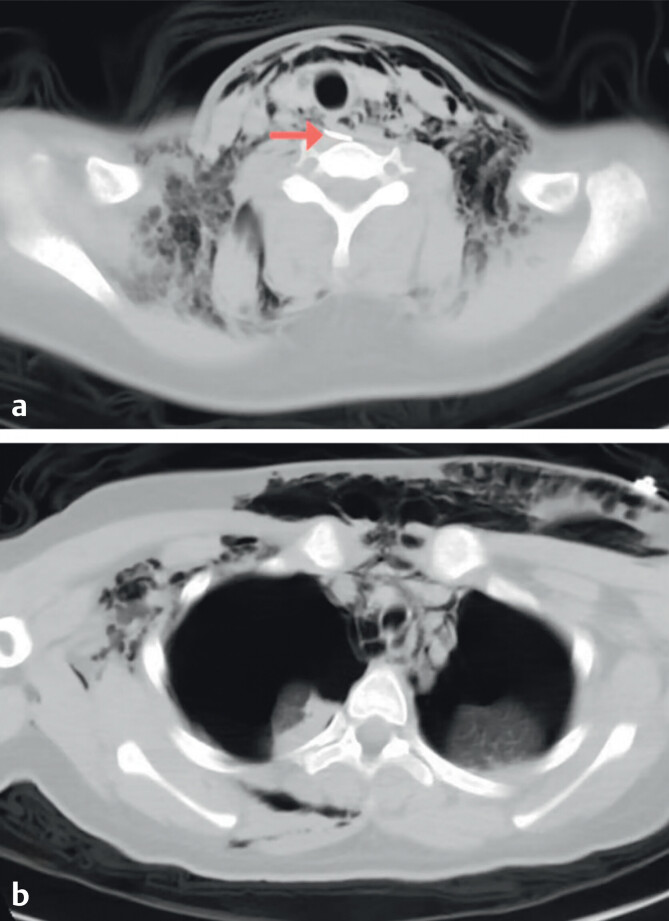
CT images. ( **a** ) A metal foreign body in the lower cervical paraesophageal region. ( **b** ) CT showed massive bilateral pneumothorax, mediastinal emphysema and extensive subcutaneous emphysema. CT, computed tomography.

The test showed marked leukocytosis. Computed tomography (CT) revealed a metal foreign body in the lower cervical paraesophageal region ( Fig. 1a ), along with massive bilateral pneumothorax, mediastinal emphysema and extensive subcutaneous emphysema ( Fig. 1b ). We immediately performed bilateral thoracic closed drainage, decompression of subcutaneous emphysema, and anti-infection therapy. Then, the endoscopic removal of blades from a complex esophagomediastinal fistula was performed ( Video 1 ).

**Video 1** Endoscopic removal of blades from a complex esophagomediastinal fistula.


**Fig. 2:**
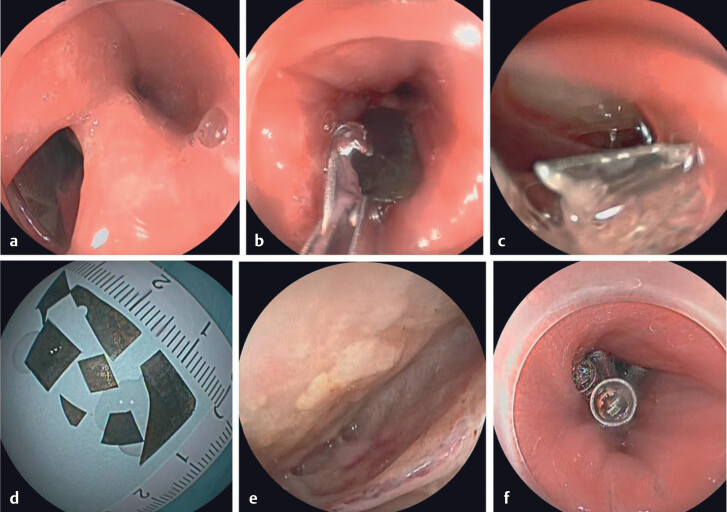
Endoscopic images. ( **a** ) Endoscopy confirmed an esophagomediastinal fistula containing multiple impacted razor blade fragments. ( **b** ) Biopsy forceps were used to grasp and remove the blade fragments. ( **c** ) A self-made magnet device was used to attract the small blade fragments successfully. ( **d** ) A total of seven blade fragments were removed. ( **e** ) No residual foreign body was found within the fistula tract. ( **f** ) The 3-cm fistulous orifice was closed with titanium clips.

Endoscopy confirmed a fistulous orifice 15 cm from incisors, communicating with mediastinum and containing multiple impacted razor blade fragments ( Fig. 2a ). The blade fragments were grasped and removed using biopsy forceps ( Fig. 2b ). A homemade device with a 25-mm round magnet successfully attracted small blade fragments. ( Fig. 2c ). Seven fragments of razor blades were removed ( Fig. 2d ). Exploration confirmed no residual foreign body within the fistula tract ( Fig. 2e ), and the 3-cm fistulous orifice was closed with 11 titanium clips ( Fig. 2f ).

**Fig. 3:**
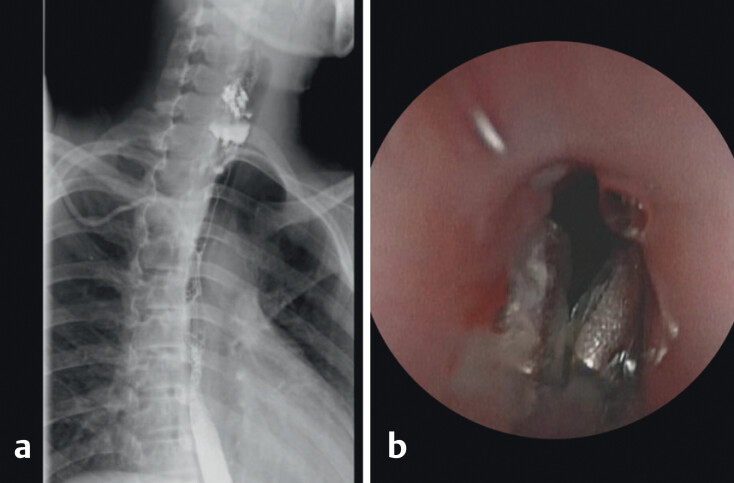
Upper gastrointestinal radiographic and endoscopic images at 2 weeks: ( **a** ) Gastrointestinal radiography showed the localized encapsulation of the persistent esophageal fistula. ( **b** ) Gastroscopy revealed satisfactory healing of the operative site with the titanium clips in place.

**Fig. 4:**
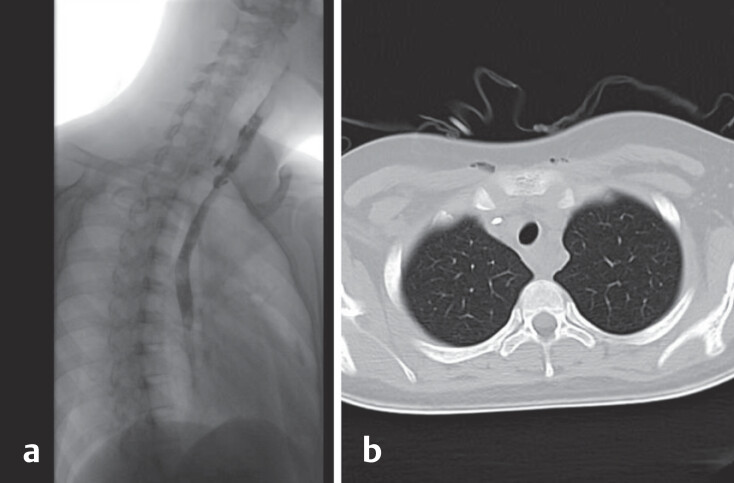
Repeat upper gastrointestinal radiography and computed tomography (CT) images at 3 weeks: ( **a** ) Gastrointestinal radiography showed that the esophageal fistula was significantly improved. ( **b** ) CT showed almost complete resolution of pneumothorax and pneumomediastinum.

The patient’s chest pain resolved after the therapy. A gastrointestinal radiography was performed at 2 weeks postoperatively, revealing the persistence of the esophageal fistula with localized encapsulation ( Fig. 3a ). Gastroscopy revealed satisfactory healing of the operative site with the titanium clips in place ( Fig. 3b ). The esophagomediastinal fistula was significantly relieved 3 weeks later ( Fig. 4a ), and CT showed almost complete resolution of pneumothorax and pneumomediastinum ( Fig. 4b ). The patient was healthy at the 2‑month follow‑up.

Acute esophageal perforation is a rare, life-threatening emergency. 1​ Endoscopic retrieval of a swallowed blade after transmural mediastinal migration is extremely rare. This case exemplified the minimally invasive concept of natural orifice transluminal endoscopic surgery, successfully managing complex extraluminal complications of esophageal perforation. 2​ ​3​ A homemade magnetic device improved procedural success and reduced iatrogenic trauma.

Endoscopy_UCTN_Code_CCL_1AB_2AD_3AF
